# 6*H*,13*H*-5,12:7,14-Dimethano­dinaphtho­[2,3-*d*:2,3-*i*][1,3,6,8]tetra­azecine

**DOI:** 10.1107/S1600536812010185

**Published:** 2012-03-14

**Authors:** Augusto Rivera, Mauricio Maldonado, Jaime Ríos-Motta, Karla Fejfarová, Michal Dušek

**Affiliations:** a Universidad Nacional de Colombia, Sede Bogotá, Facultad de Ciencias, Departamento de Química, Grupo de Investigación Síntesis de Heterociclos, Cra 30 No.45-03, Bogotá, Código Postal 111321, Colombia; b Institute of Physics ASCR, v.v.i., Na Slovance 2, 182 21 Praha 8, Czech Republic

## Abstract

In the title compound, C_24_H_20_N_4_, obtained through the condensation of naphthalene-2,3-diamine with formaldehyde in methanol, the mol­ecule is located on a special position of site symmetry -4. Due to symmetry considerations, the aromatic rings are strictly perpendicular to each other. In the crystal, mol­ecules are linked by pairs of C—H⋯π inter­actions into columns along [110].

## Related literature

For chemical background to the synthesis of the title compound, see: Volpp (1962 ▶). For related structures, see: Murray-Rust & Smith (1975 ▶); Rivera *et al.* (2009 ▶, 2011 ▶). For bond-length data, see: Allen *et al.* (1987 ▶).
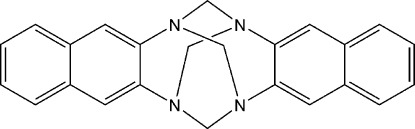


## Experimental

### 

#### Crystal data

C_24_H_20_N_4_
*M* *_r_* = 364.5
Tetragonal, 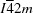

*a* = 7.1996 (2) Å
*c* = 17.4511 (5) Å
*V* = 904.56 (6) Å^3^
*Z* = 2
Cu *K*α radiation
μ = 0.63 mm^−1^
*T* = 120 K
0.45 × 0.22 × 0.15 mm

#### Data collection

Agilent Xcalibur diffractometer with an Atlas (Gemini ultra Cu) detector
Absorption correction: multi-scan (*CrysAlis PRO*; Agilent, 2010 ▶) *T*_min_ = 0.50, *T*_max_ = 0.90
4873 measured reflections
273 independent reflections
268 reflections with *I* > 3σ(*I*)
*R*_int_ = 0.025

#### Refinement

*R*[*F* ^2^ > 2σ(*F* ^2^)] = 0.027
*wR*(*F* ^2^) = 0.077
*S* = 1.85
273 reflections
51 parameters
Only H-atom coordinates refined
Δρ_max_ = 0.08 e Å^−3^
Δρ_min_ = −0.15 e Å^−3^

### 

Data collection: *CrysAlis PRO* (Agilent, 2010 ▶); cell refinement: *CrysAlis PRO*; data reduction: *CrysAlis PRO*; program(s) used to solve structure: *SIR2002* (Burla *et al.*, 2003 ▶); program(s) used to refine structure: *JANA2006* (Petříček *et al.*, 2006 ▶); molecular graphics: *DIAMOND* (Brandenburg & Putz, 2005 ▶); software used to prepare material for publication: *JANA2006*.

## Supplementary Material

Crystal structure: contains datablock(s) global, I. DOI: 10.1107/S1600536812010185/bg2445sup1.cif

Structure factors: contains datablock(s) I. DOI: 10.1107/S1600536812010185/bg2445Isup2.hkl

Supplementary material file. DOI: 10.1107/S1600536812010185/bg2445Isup3.cml

Additional supplementary materials:  crystallographic information; 3D view; checkCIF report

## Figures and Tables

**Table 1:** Hydrogen-bond geometry (Å, °) *Cg*1 and *Cg*2 are the centroids of the C2–C4/C2′–C4′ and C4–C6/C4′–C6′ rings, respectively.

*D*—H⋯*A*	*D*—H	H⋯*A*	*D*⋯*A*	*D*—H⋯*A*
C3—H3⋯*Cg*2^i^	1.042 (18)	2.648 (14)	3.6921 (14)	178.2 (15)
C5—H5⋯*Cg*1^i^	1.047 (18)	2.652 (14)	3.6979 (14)	178.0 (16)

Symmetry code: (i) 
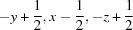
.

**Fig. 1.**
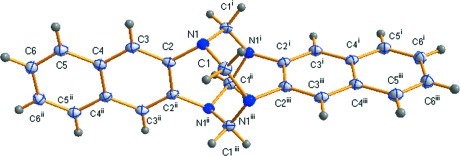
A view of (I) with the numbering scheme, displacement ellipsoids are drawn at the 50% probability level. Symmetry codes: (i) 1 + y,1 - x,-z; (ii) 2 - x,-y,z; (iii) 1 - y,-1 + x,-z.

**Fig. 2.**
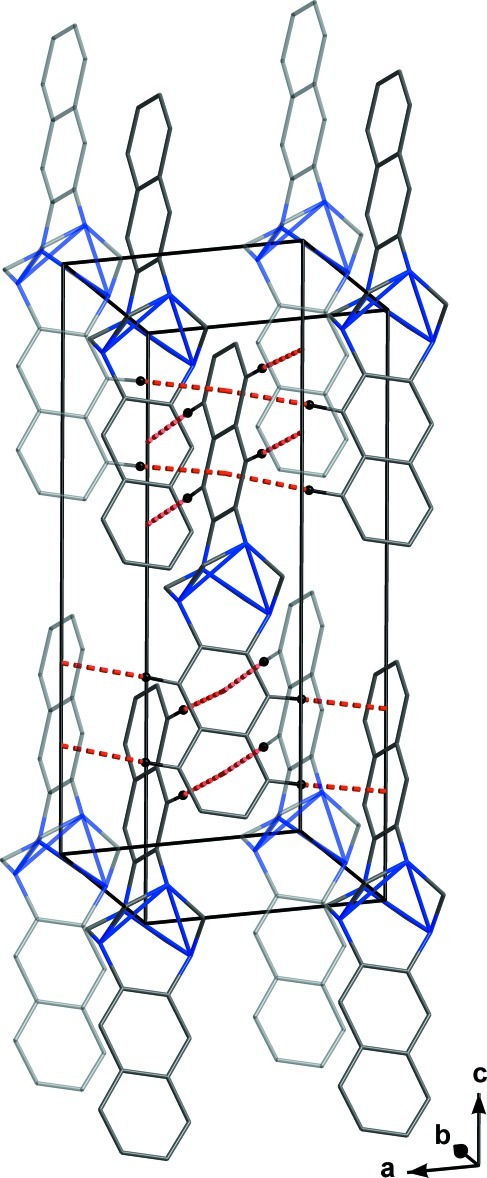
Packing of the molecules of the title compound view along b axis.

## supplementary crystallographic information

### Comment

We have as a general goal the design and synthesis of new macrocyclic saturated ring-fused aminals, of considerable interest as useful intermediates for the synthesis of *N*-containing heterocyclic compounds. These aminals comprise a family of preformed electrophilic reagents which have been utilized in condensation reactions with electron-rich aromatic compounds in a variant of the Mannich reaction. These ring-fused aminals are frequently prepared by reaction of 1,2-diamines with formaldehyde (Volpp, 1962). By an analogous route we prepared for the first time 6*H*,13*H*-5,12:7,14-dimethanodinaphtho[2,3-*d*:2,3-*i*][1,3,6,8]tetraazecine (I).

The molecular structure and atom-numbering scheme for (I) are shown in Fig. 1. Unlike the related structures, which crystallized in orthorhombic space groups *Ab*a2 (Rivera *et al.*, 2009, 2011) and P*bcn* (Murray-Rust & Smith, 1975), the title compound (I) crystallizes in the tetragonal I42*m* space group with one quarter-molecule in the asymmetric unit (located on a special position of site symmetry 4). The X-ray structure of I shows similar features to other ring-fused aminals. So, the bond lengths and angles are normal (Allen *et al.*, 1987) and similar to those observed for related structures (Murray-Rust & Smith, 1975; Rivera *et al.*, 2009; Rivera *et al.*, 2011).

Due to symmetry considerations the aromatic rings are strictly perpendicular to each other. In the crystal packing (Fig. 2), the molecules are linked by a pair of C—H···π interactions (Table 1) into columns along [110].

### Experimental

A solution of naphthalene-2,3-diamine (158 mg, 1 mmol) in methanol (10 ml) was added dropwise at 273 K to 5 ml of 37% aqueous formaldehyde. The reaction mixture was stirring at this temperature for 1 h and its completion was monitored by TLC. After completion, the contents were poured over cold water (10 ml). The resultant solid was isolated by filtration, washed with cold water, dried in vacuum and recrystallized from ethyl acetate to give the title compound with 28% yield. The melting point of the title structure is 484 K.

^1^H NMR (δ, 400 MHz, CDCl_3_): 4.55, 7.52, 7.74, 7.86. ^13^C NMR (δ, 100 MHz, CDCl_3_): 70.2, 125.6, 126.2, 127.7, 133.0, 151.9.

### Refinement

The H atoms atoms were found in difference Fourier maps and their coordinates were refined freely. The isotropic atomic displacement parameters of hydrogen atoms were evaluated as 1.2×*U*_eq_ of the parent atom. As the structure contains only light atoms, the Friedel-pair reflections were merged and the Flack parameter has not been determined.

### Crystal data

**Table:**

C_24_H_20_N_4_	*D*_x_ = 1.338 Mg m^−^^3^
*M**_r_* = 364.5	Cu *K*α radiation, λ = 1.5418 Å
Tetragonal, *I*42*m*	Cell parameters from 3581 reflections
Hall symbol: I -4 2	θ = 5.1–67.0°
*a* = 7.1996 (2) Å	µ = 0.63 mm^−^^1^
*c* = 17.4511 (5) Å	*T* = 120 K
*V* = 904.56 (6) Å^3^	Irregular shape, yellow
*Z* = 2	0.45 × 0.22 × 0.15 mm
*F*(000) = 384	

### Data collection

**Table:**

Agilent Xcalibur diffractometer with an Atlas (Gemini ultra Cu) detector	273 independent reflections
Radiation source: Enhance Ultra (Cu) X-ray Source	268 reflections with *I* > 3σ(*I*)
Mirror monochromator	*R*_int_ = 0.025
Detector resolution: 10.3784 pixels mm^-1^	θ_max_ = 67.1°, θ_min_ = 5.1°
Rotation method, ω scans	*h* = −8→8
Absorption correction: multi-scan (*CrysAlis PRO*; Agilent, 2010)	*k* = −8→8
*T*_min_ = 0.50, *T*_max_ = 0.90	*l* = −20→20
4873 measured reflections	

### Refinement

**Table:**

Refinement on *F*^2^	4 constraints
*R*[*F*^2^ > 2σ(*F*^2^)] = 0.027	Only H-atom coordinates refined
*wR*(*F*^2^) = 0.077	Weighting scheme based on measured s.u.'s *w* = 1/(σ^2^(*I*) + 0.0016*I*^2^)
*S* = 1.85	(Δ/σ)_max_ = 0.003
273 reflections	Δρ_max_ = 0.08 e Å^−^^3^
51 parameters	Δρ_min_ = −0.15 e Å^−^^3^
0 restraints	

### Special details

**Table:**

Refinement. The refinement was carried out against all reflections. The conventional *R*-factor is always based on *F*. The goodness of fit as well as the weighted *R*-factor are based on *F* and *F*^2^ for refinement carried out on *F* and *F*^2^, respectively. The threshold expression is used only for calculating *R*-factors *etc*. and it is not relevant to the choice of reflections for refinement.The program used for refinement, Jana2006, uses the weighting scheme based on the experimental expectations, see _refine_ls_weighting_details, that does not force *S* to be one. Therefore the values of *S* are usually larger than the ones from the *SHELX* program.

### Fractional atomic coordinates and isotropic or equivalent isotropic displacement parameters (Å^2^)

**Table:**

	*x*	*y*	*z*	*U*_iso_*/*U*_eq_	
N1	0.86029 (13)	0.13971 (13)	0.04360 (8)	0.0180 (4)	
C1	0.7560 (2)	0	0	0.0187 (4)	
C2	0.92982 (16)	0.07018 (16)	0.11529 (9)	0.0175 (4)	
C3	0.86239 (18)	0.13761 (18)	0.18326 (11)	0.0194 (4)	
C4	0.9299 (2)	0.0701 (2)	0.25428 (9)	0.0181 (4)	
C5	0.86279 (18)	0.13721 (18)	0.32562 (11)	0.0221 (4)	
C6	0.93073 (18)	0.06927 (18)	0.39302 (9)	0.0222 (4)	
H1	0.677 (2)	0.0686 (18)	−0.0400 (7)	0.0225*	
H3	0.760 (2)	0.240 (2)	0.1798 (11)	0.0233*	
H5	0.760 (2)	0.240 (2)	0.3243 (12)	0.0265*	
H6	0.886 (2)	0.114 (2)	0.4421 (13)	0.0266*	

### Atomic displacement parameters (Å^2^)

**Table:**

	*U*^11^	*U*^22^	*U*^33^	*U*^12^	*U*^13^	*U*^23^
N1	0.0174 (5)	0.0174 (5)	0.0191 (8)	0.0016 (7)	0.0002 (4)	−0.0002 (4)
C1	0.0152 (8)	0.0201 (8)	0.0210 (7)	0	0	0.0003 (7)
C2	0.0158 (5)	0.0158 (5)	0.0208 (9)	0.0001 (7)	−0.0016 (5)	0.0016 (5)
C3	0.0174 (6)	0.0174 (6)	0.0235 (10)	0.0026 (7)	0.0007 (5)	−0.0007 (5)
C4	0.0168 (6)	0.0168 (6)	0.0208 (8)	−0.0015 (7)	−0.0008 (5)	0.0008 (5)
C5	0.0211 (6)	0.0211 (6)	0.0240 (10)	0.0031 (8)	0.0002 (4)	−0.0002 (4)
C6	0.0234 (6)	0.0234 (6)	0.0196 (7)	0.0012 (8)	0.0007 (5)	−0.0007 (5)

### Geometric parameters (Å, º)

**Table:**

N1—C1	1.4680 (13)	C3—H3	1.042 (18)
N1—C1^i^	1.4680 (13)	C4—C4^iii^	1.428 (2)
N1—C2	1.437 (2)	C4—C5	1.420 (2)
C1—H1	1.027 (13)	C5—C6	1.365 (2)
C1—H1^ii^	1.027 (13)	C5—H5	1.047 (18)
C2—C2^iii^	1.4292 (17)	C6—C6^iii^	1.4106 (18)
C2—C3	1.370 (2)	C6—H6	0.97 (2)
C3—C4	1.417 (2)		
			
C1—N1—C1^i^	115.64 (9)	C2—C3—C4	120.94 (12)
C1—N1—C2	113.00 (8)	C2—C3—H3	116.7 (11)
C1^i^—N1—C2	113.00 (8)	C4—C3—H3	122.3 (11)
N1—C1—N1^iv^	118.44 (11)	C3—C4—C4^iii^	118.99 (14)
N1—C1—H1	107.8 (7)	C3—C4—C5	122.26 (13)
N1—C1—H1^ii^	105.1 (7)	C4^iii^—C4—C5	118.74 (14)
N1^iv^—C1—H1	105.1 (7)	C4—C5—C6	120.80 (13)
N1^iv^—C1—H1^ii^	107.8 (7)	C4—C5—H5	117.5 (11)
H1—C1—H1^ii^	112.7 (11)	C6—C5—H5	121.7 (11)
N1—C2—C2^iii^	119.50 (13)	C5—C6—C6^iii^	120.46 (14)
N1—C2—C3	120.43 (11)	C5—C6—H6	121.7 (10)
C2^iii^—C2—C3	120.06 (14)	C6^iii^—C6—H6	117.8 (10)

Symmetry codes: (i) *y*+1, −*x*+1, −*z*; (ii) *x*, −*y*, −*z*; (iii) −*x*+2, −*y*, *z*; (iv) −*y*+1, *x*−1, −*z*.

### Hydrogen-bond geometry (Å, º)

Cg1 and Cg2 are the centroids of the C2–C4/C2'–C4' and C4–C6/C4'–C6' rings, respectively.

**Table:**

*D*—H···*A*	*D*—H	H···*A*	*D*···*A*	*D*—H···*A*
C3—H3···*Cg*2^v^	1.042 (18)	2.648 (14)	3.6921 (14)	178.2 (15)
C5—H5···*Cg*1^v^	1.047 (18)	2.652 (14)	3.6979 (14)	178.0 (16)

Symmetry code: (v) −*y*+1/2, *x*−1/2, −*z*+1/2.
